# Consequences of the COVID-19 Pandemic on the Incidence, Management and Outcomes of *Staphylococcus aureus* Bacteraemia: Experience in a Spanish Hospital

**DOI:** 10.3390/pathogens13100847

**Published:** 2024-09-29

**Authors:** Elizabeth Lorenzo-Hernández, Francisco Rivas-Ruiz, Alfonso Del Arco-Jiménez

**Affiliations:** 1 Internal Medicine Department, Costa del Sol University Hospital, A-7, Km 187, 29603 Marbella, Malaga, Spain; 2 Research Unit, Costa del Sol University Hospital, A-7, Km 187, 29603 Marbella, Malaga, Spain; francisco.rivas.ruiz.sspa@juntadeandalucia.es

**Keywords:** *Staphylococcus aureus*, bacteraemia, COVID-19, mortality, complications, adherence, bundle, nosocomial

## Abstract

This work aims to assess the impact of the COVID-19 pandemic on the mortality and incidence of complications in patients with bacteraemia due to *Staphylococcus aureus* (BSA). All episodes of BSA at the Costa del Sol University Hospital (Marbella, Spain) were recorded during the acute phase of the COVID-19 pandemic (March 2020–March 2022) and compared with those in a previous period (February 2018–February 2020). Demographic, clinical and prognostic variables were recorded. The outcomes were measured as 14- and 30-day mortality and the incidence of complications/death. Mortality during the pandemic was 28.7% at 14 days and 35% at 30 days, while in the pre-pandemic group, it was 18.9% and 23.3%, respectively. For overall complications/deaths, the incidence rate was higher in the pandemic group, with 42.7%. No significant differences were observed between groups. Seventeen patients with COVID-19 were identified, with mortality rates of 64.7% and 70.6% at 14 and 30 days. Multivariate analysis established the presence of sepsis at diagnosis as a predictor of mortality, but not BSA, during the pandemic phase. In conclusion, BSA is a disease with high mortality, which was slightly higher during the pandemic phase. No differences were found in adherence to the bundle in our centre.

## 1. Introduction

COVID-19, a primarily respiratory disease caused by the SARS-CoV-2 virus, has caused a social crisis and a challenge for science and healthcare systems, forcing us to change the way we understand medical practice in a context of uncertainty, stress, the saturation of healthcare services, a new disease, etc. Furthermore, it has affected the way in which multiple diseases are addressed due to their contagiousness and the morbidity and mortality they have produced.

On the other hand, bacteraemia due to *Staphylococcus aureus* (BSA) is very common in our environment, with around 50 cases/100,000 person-years, and involves significant morbidity and mortality, with a mortality rate of around 20–30% [1,2,3,4,5,6]. This is the bacteraemia with the highest mortality rate in global figures [7]. It has been the subject of study on multiple occasions, with the aim of developing packages of measures or bundles to unify the way of approaching this entity and thus reduce mortality [8,9]. Despite this, however, there is still no uniformity in the way this entity is approached, with variability existing among professionals. The main factors that influence mortality are age and the presence of sepsis at diagnosis, as well as the early choice of an appropriate antibiotic, and not so much the virulence and resistance characteristics of the bacteria [5,10]. The way that BSA occurs varies, which makes it difficult to understand. The clinical incidence may correspond to various sources of infection that cause bacteraemia or to the consequences of bacteraemia (for example, remote embolisms).

This difficulty in identifying BSA increased during the COVID-19 pandemic period, given that it is a disease that has been incorporated into the differential diagnosis as an infectious process, delaying its diagnosis and treatment. There are studies on BSA that maintain that the difference in mortality rates between centres could be due to different ways of caring for patients, so the management of bacteraemia in each centre could have been different in the face of a health crisis situation, since this represents a relevant change in normal practice [6].

As with other respiratory viral infections, there is a predisposition to bacterial and fungal co-infection for several reasons: parenchymal destruction, cytokine storm and simultaneous defects in both innate and acquired immune responses that provide a favourable environment for bacterial growth and adherence. Manna S. et al. have provided a brilliant review on the molecular pathogenesis of secondary bacterial infections associated with viral infections, including SARS-CoV-2 [11]. Unlike infection by the influenza virus, also associated with bacterial co-infection, there is a low proportion of community acquisition of the disease. Due to their serious condition, COVID-19 patients are in closer contact with healthcare personnel, receive care (including being placed in a prone position), undergo interventions such as catheterisations or receive immunosuppressive drugs. *S. aureus* is one of the most frequently isolated microorganisms in blood cultures in patients with COVID-19 [12,13,14,15,16,17] and is the most frequently isolated in respiratory samples in patients in intensive care [18,19]. Higher mortality has been described in patients who have suffered from COVID-19, especially when they are admitted to the intensive care unit or undergo mechanical ventilation [16,20,21,22].

Antimicrobial Stewardship Programmes aim to improve the prognosis of infections. They were affected by the COVID-19 pandemic due to the organisational changes that this infection brought about in hospitals and modifications to the way they care for their patients. On the one hand, there are studies that claim that the implementation of measures to reduce the transmission of infections (emphasis on hand hygiene, restrictions on visits, extensive disinfection of certain areas, use of masks, etc.) have reduced the transmission of infections and, with them, the incidence of bacteraemia due to *S. aureus* or infection by *Clostridioides difficile* [23]. On the other hand, other studies show that the incidence rate is higher [16]. The information coming from studies regarding the impact of the pandemic on the incidence, management and outcome of this bacteraemia is heterogeneous, which is why the present study has been performed to help increase the knowledge regarding its mortality and complications, with the aim of improving care for these patients.

The aim of this study was to assess the impact of the acute phase of the COVID-19 pandemic on mortality and the incidence of complications in adult patients with BSA by comparing these outcomes with those in a period prior to this phase.

## 2. Materials and Methods

### 2.1. Study Design

This was an observational and retrospective study including non-paediatric patients (≥14 years) with bacteraemia documented by blood culture for *S. aureus* included in the bacteraemia registry of our centre (Costa del Sol University Hospital, Marbella, Malaga, Spain) during the acute phase period of the COVID-19 pandemic in Andalusia (according to the COVID-19 report in Andalusia) [24].

The data in this period were compared with those from a period of equal duration, established between 1 February 2018 and 29 February 2020. Demographic, clinical and prognostic variables were collected from digital medical records. Cases with persistent bacteraemia were considered as a single patient.

This study was approved by the Costa del Sol Research Ethics Committee with the code 004_dic23-PI on 21 December 2023.

### 2.2. Participants

Inclusion criteria were all patients older than 14 years treated at the Costa del Sol University Hospital in Marbella (Malaga, Spain) who developed *S. aureus* bacteraemia from 1 March 2020 to 31 March 2022, regardless of previous comorbidities. All patients aged less than 14 years, usually treated by the paediatric department, and patients who did not complete treatment at our centre or in the public hospital network of Andalusia were excluded.

### 2.3. Definitions

Healthcare-related acquisition was defined as a case in which patients were in a nursing home or attended a dialysis centre or outpatient clinic, and nosocomial acquisition was that in which bacteraemia occurred more than 48 h after admission. Patients were evaluated using the Charlson index to determine previous comorbidities, with the Pitt bacteraemia index and the MacCabe and Jackson index used as prognostic indices. We used the McCabe and Jackson criteria initially described in 1962. The classic McCabe–Jackson criteria for predicting the likelihood of survival of patients with Gram-negative bacteraemia on the basis of the level of underlying disease was first developed based on observations of endotoxin tolerance in humans [25]. In healthcare-associated infection (HAI) point prevalence surveys, it is used as a subjective score of underlying illness severity. This simple method classifies patients according to a prognosis of rapidly fatal (<1 year), ultimately fatal (1–4 years) and non-fatal (>5 years).

The source of infection was defined by clinical data collected from the medical history, by microbiological criteria (isolation of *S. aureus* in other clinical samples) or by both. Antibiotic treatment was classified as empirical or directed based on whether the prescription was made before or after the results of the blood cultures were known, respectively, and the adequacy of the treatment in terms of the spectrum coverage and dosage was assessed according to the latest guidelines for the treatment of bacteraemia [26,27]. Compliance with the bundle was defined as an adequate duration of treatment, the performance of control blood cultures 48–72 h after starting appropriate treatment and the performance of an echocardiogram in the case of clinical indications (individual risk, persistent bacteraemia, complicated bacteraemia, community acquisition or unknown cause) [4]. Those who had received an adequate duration of treatment based on the underlying clinical process and individual risk and who had undergone echocardiography and control blood cultures were identified. Persistent bacteraemia was defined as that in which *S. aureus* was isolated from control blood cultures 48–72 h after starting appropriate treatment. The appropriate treatment duration was considered to be 14 days for uncomplicated bacteraemia or longer, depending on whether the bacteraemia was complicated or whether the cause or complications arising from the bacteraemia required a longer duration.

### 2.4. Microbiological Identification Techniques

Microbiological identification was performed directly from the positive blood culture using mass spectrometry (MALDI-TOF, Bruker Daltonics, Billerica, MA, USA). Following the identification of *S. aureus*, a polymerase chain reaction (PCR) was performed directly on the positive blood culture for the early detection of methicillin resistance (mecA/mecC) (BD MAX StaphSR Assay (SR assay); BD, Franklin Lakes, NJ, USA). Antimicrobial susceptibility was determined using the turbidimetric method (Vitek 2, Biomérieux, Marcy-l’Étoile, France). The detection of *S. aureus* in other clinical samples (catheters, respiratory samples, etc.) was also performed using MALDI-TOF on the culture. SARS-CoV-2 detection was performed using multiple commercial PCR kits on nasopharyngeal swab samples.

### 2.5. Main Study Variables

The main variables to determine the impact of the COVID-19 pandemic on mortality due to BSA were mortality at 14 and 30 days from the first positive blood culture, the presence of sepsis at diagnosis, the development of complications (defined as endocarditis, osteoarticular, myositis, aortitis, lung abscess, central nervous system abscess, abscess in another location or others), the presence of persistent bacteraemia and adherence to the measures recommended in the bundle for BSA.

### 2.6. Statistical Analysis

A descriptive analysis was performed using measures of central tendency, dispersion and position for quantitative variables and frequency distribution for qualitative variables. For each of the dichotomous outcome variables of interest, bivariate analysis was performed using the Chi-square test (or Fisher’s exact test) for qualitative independent variables and the Student’s t-test (or Mann–Whitney U test in the case of non-normal distribution) for quantitative independent variables. A multivariate logistic regression model was then constructed for the outcome variables (mortality at 14 and 30 days, together with the combined variable presence of mortality or complications at 30 days) using the forward stepwise method with the independent variables of interest (group, age, sex and Charlson index), and odds ratios (ORs) with their respective 95% confidence intervals were determined. In the different analyses, the level of statistical significance was established at *p* < 0.05. SPSS v28 software was used.

## 3. Results

A total of 31,820 hospital admissions for any cause were recorded in adults during the acute phase of the pandemic, hereinafter referred to as the pandemic period, and 34,371 admissions were recorded in the pre-pandemic period. A total of 212 episodes of bacteraemia were identified in the study period, 122 corresponding to the pandemic phase group and 90 episodes to the comparison group. The risk of developing BSA during the pandemic period was 3.83 per 1000 hospital admissions, and in the pre-pandemic period, it was 2.62 per 1000 admissions (*p* = 0.09).

Demographic and clinical variables are described in Table 1. Males predominated, with 69.7% and 74.4% in the pandemic and pre-pandemic groups, respectively. The mean ages (±standard deviation) in the pandemic and pre-pandemic periods were 66 (±15) and 63 (±17), respectively. There were no significant differences in comorbidities between the two groups. More than half of the infections in the pandemic group were of nosocomial origin (54.1%), which is statistically significant. Regarding the cause of infection, the two most frequent groups in the pandemic group were skin and soft tissue infection (SSTI) and respiratory origin, without reaching statistical significance in the latter case. In the pre-pandemic group, the most frequent causes were catheter-associated infection and SSTI. Primary bacteraemia cases were 12.3% of all bacteraemia cases in the pandemic phase and 16.7% in the pre-pandemic phase, with no differences between groups.

Regarding the outcome of bacteraemia, in the pandemic phase group, there were mortality rates of 28.7% at 14 days and 35% at 30 days, while in the pre-pandemic group, they were 18.9% and 23.3%, respectively. In the overall complications/death, the incidence rate was higher in the pandemic group at 42.7% of patients. However, no significant differences were obtained between the two groups (Table 2).

Of the variables analysed in the multivariate model, a relationship was found between occurrence of the complication/death complex with sepsis at diagnosis (*p* < 0.001), with an OR of 4.96. The risk of incidence of the complication/death complex in relation to age is *p* = 0.06. There were no differences between groups regarding the presence of complications/death.

Regarding adherence to the measures recommended by the antimicrobial stewardship consultancy and the degree of compliance with the bundle, no significant differences were found between groups (Table 3).

### Bacteraemia by Staphylococcus aureus in Patients with COVID-19

Seventeen patients with COVID-19 were identified in the pandemic group (13.95% of the total) (Table 4). Infection acquisition was mostly nosocomial, at 82.4%. The most frequent focus was respiratory (52.9% of cases). Regarding 30-day mortality, five deaths could be attributed to COVID-19 and five to BSA, with data missing for two patients.

## 4. Discussion

This study investigated the outcome of BSA in the Costa del Sol University Hospital in Marbella (Malaga, Spain) during the COVID-19 pandemic period, with the aim of clarifying whether this health crisis led to an increase in the mortality or incidence of complications of this bacteraemia, as well as to elucidate whether there were changes in its incidence and management during the study period. It represents the work with the largest sample size compared to other studies with similar objectives [10].

The incidence of BSA was slightly higher during the pandemic period, although no significant differences were found when compared to the pre-pandemic period. This incidence is variable in the literature; on the one hand, there are studies that found a higher incidence of both community-acquired and nosocomial BSA during the pandemic (attributed to the delay in treatment, the severity of COVID-19 patients, the widespread use of antibiotic therapy or greater exposure to invasive procedures or lower use of infection control measures) [17,20,28]; on the other hand, other studies found a significantly lower incidence of nosocomial BSA during the pandemic, attributing it to the reduced contact of the patient with staff, the restriction of visits, the use of masks and the decontamination of surfaces [23].

In both groups, there is a predominance of male patients, slightly higher in the group from the acute phase of the pandemic, but with the same comorbidity burden according to the Charlson index and the same severity according to the Pitt score. The patients included in the sample have similar characteristics with regard to other multi-centre studies, such as an age of around 65 years and a median comorbidity, calculated by the Charlson index, of 2 [6]. We found a low prevalence of methicillin-resistant *S. aureus* (MRSA) in our centre, 7%, somewhat lower than in other Spanish centres [12] and similar to that in other European studies [10]. There was a slight increase in cases of MRSA bacteraemia during the pandemic period, although without statistically significant differences, probably due to the sample size. Other authors have found an increase in MRSA bacteraemia during the pandemic, which is attributed to the practically universal use of antibiotic therapy in their patients with COVID-19 pneumonia [5].

The distribution of the causes of BSA between both groups is similar to that described by other authors, with a non-significant increase in the respiratory focus in the pandemic group, consistent with other series [12], even above other more frequent foci of infection in the literature, such as catheter-associated infection [10]. There is a lower representation of primary BSA in both groups compared to other series—around 20–40% (2.6)—which could be related to the insistence on the investigation of cases in both study periods by the antimicrobial stewardship consultancy after learning the positive result of the blood culture.

Regarding the form of acquisition of BSA, in our centre, nosocomial acquisition was 54.1% in the pandemic phase, with significant differences when compared to the pre-pandemic phase (38.9% of cases). There is heterogeneity among studies regarding hospital or nosocomial acquisition, ranging between 45 and 70% [6,10].

In the periods studied, there was an increase in mortality at 14 and 30 days in the pandemic period, as well as in the global deaths/complications, but the change was not significant. In addition, 30-day mortality was slightly higher (35%) than in most studies, which have established it between 20 and 30% in non-specific periods of the COVID-19 pandemic [1,2,3,4,5,6]. This mortality rate was surpassed during the pandemic period by a hospital in Tehran (Iran) with 41.9% mortality [5], and only in this centre did they describe a lower incidence of mortality in non-COVID-19 patients than previously reported, which they related to a small sample size and a comparison with an older national cohort [14]. In another work reported by Böing et al., changes in the prognosis were observed, and although it had a smaller sample size, they related it to good adherence to the bundle [10]. The incidence of complications arising from bacteraemia has not been studied in any work. In the literature, the main causes of mortality associated with BSA are a respiratory focus and primary bacteraemia [3,6]. In our study, the slight increase in mortality was accompanied by an increase in respiratory causes and a decrease in primary BSA, but both without significant differences. The logistic regression model used also did not find significant differences for the variables studied, probably due to the sample size.

In our centre, each bacteraemia case reported by the microbiology laboratory is communicated to the antimicrobial stewardship expert. It is the task of the expert to locate the doctor responsible for the patient, inform him/her and make recommendations on how to proceed, mainly verbally and occasionally in writing, or to locate the patient at home if he/she has been discharged from the emergency department. This means that all cases of bacteraemia have been advised by the antimicrobial stewardship expert, except those with an early fatal outcome. This continued during the acute phase of the pandemic despite the overload of care facilities. Several studies highlight the role of the infectious disease consultant in improving the prognosis of this disease [2,3,4]. In some centres, there was less consultation and less adherence to recommendations during the pandemic period [9]. However, in our study, observed adherence was around 50% in both periods for compliance with all measures recommended in the bundle. Adherence was slightly higher in the pandemic group, but not significantly, increasing from 50.7% to 54.5%, similar to other studies with a retrospective nature [29]. This greater adherence may be due to greater awareness regarding disease management after each contact with the antimicrobial stewardship team; however, we think that the lack of generalisation of written recommendations in a place with a high turnover of responsible physicians (on-call, consultations, etc.) could have had a significant impact on overall adherence to the measures in the bundle in our centre. In other studies [8], adherence to the bundle was higher, probably because they were prospective or quasi-experimental studies and did not deal with real-life data, although there have also been prospective studies with low adherence, around 18%, which related this low adherence, despite being a prospective study, to the heterogeneity of the participating centres, with a worldwide distribution [30].

### Bacteraemia Caused by Staphylococcus aureus in Patients with COVID-19

Of our patients, 17 suffered from COVID-19, with mortality at 14 and 30 days of 64.7% and 70.6%, respectively, practically double the mortality of patients who did not suffer from it, much higher and significantly higher than in other studies with similar population, which also did not reveal differences from patients without COVID-19 [12]. However, this is probably an over-represented mortality rate due to the small sample size. The increased mortality compared to other patients with BSA is supported by other studies where it has been found that co-infected patients have higher mortality, as well as a higher incidence rate of shock and respiratory failure. These mortality data are explained by nosocomial acquisition, the use of immunosuppressive drugs, the interventionism to which they were subjected, with greater difficulty in practising nursing procedures due to contact precautions, or the characteristics of the virus [13,14,15,16].

The main form of acquisition is nosocomial, as in other works, and the source of infection is usually also respiratory [12,31], except in the work of Mason [17], where catheter-associated bacteraemia was more frequent than a respiratory origin of the infection, which is related to the change in the staff/patient ratio and the interventionism to which they were subjected.

## 5. Limitations

The main limitations of this study were its single-centre nature and retrospective design. It should be noted that the nature of the design did not include the calculation of a sample size for the detection of differences in both groups, so the differences may be underestimated. Furthermore, mortality attributable to bacteraemia or other causes, including COVID-19, was interpreted based on information collected from the medical history and was often of unknown origin.

This study had a publication delay due to the impossibility of extracting the data until definitive information was available in the digital clinical history of our hospital, which has a 1-year lag.

## 6. Conclusions

The severity and complexity of BSA require a group of experts in each hospital to be dedicated to advising on and monitoring this process, preferably at the bedside. Health emergency situations put these measures in jeopardy, as in the case of the COVID-19 pandemic, where efforts were redirected to the care of these patients. Although a trend was observed, no statistically significant differences were found in terms of mortality or complications in patients treated for BSA during the COVID-19 pandemic. New studies with a larger sample size are necessary to analyse these data, as well as the risk factors that could explain the worse prognosis.

## Figures and Tables

**Table 1:** Demographic and clinical characteristics.

		Pre-Pandemic Period	Pandemic Period	*p*
Sex (male), *n* (%)		67 (74.4)	85 (69.7)	0.54
Age, mean years (SD)		63 (17.1)	66.3 (15.5)	0.21
Spanish nationality		62 (68.9%)	75 (61,5%)	0.33
Charlson index, median (P25–P75)		2 (1–4.3)	2 (1–3.3)	0.03
Pitt score, median (P25–P75)		1 (0–2)	0 (0–3)	0.08
McCabe, *n* (%)	Not fatal	33 (36.7)	49 (40.2)	0.13
	Quickly fatal	25 (27.8)	46 (37.7)	
	Ultimately fatal	32 (35.6)	27 (22.1)	
Bacteria, *n* (%)	MSSA	85 (94.4)	114 (92.6)	0.80
	MRSA	5 (5.6)	9 (7.4)	
Acquisition, *n* (%)	Community	29 (32.2)	31 (25.4)	-
	Related to healthcare	26 (28.9)	25 (20.5)	
	Nosocomial	35 (38.9)	66 (54.1)	0.04
Source of infection, *n* (%)	Catheter	30 (33.3)	25 (20.5)	0.05
	SSTI	20 (22.2)	31 (25.4)	-
	Respiratory	12 (13.3)	26 (21.3)	0.19
	Endocarditis	4 (4.4)	8 (6.6)	-
	Osteoarticular	4 (4.4)	4 (3.3)	
	Abdominal	1 (1.1)	7 (5.7)	
	Urinary	3 (3.3)	5 (4.1)	
	Others	1 (1.1)	1 (0.8)	
	Primary	15 (16.7)	15 (12.3)	0.48

*n* total number of patients in that category. SD: standard deviation. P25: 25th percentile. P75: 75th percentile. MSSA: methicillin-sensitive *S. aureus*. MRSA: methicillin-resistant *S. aureus*. SSTI: skin and soft tissue infection.

**Table 2:** Outcomes.

		Pre-Pandemic Period	Pandemic Period	*p*
14-day mortality, *n* (%)		17/90 (18.9)	35/122 (28.7)	0.14
30-day mortality, *n* (%)		21/88 (23.3)	42/120 (35)	0.09
Complications/death at 30 days, *n* (%)		29/88 (33)	50/120 (41.7)	0.25
Complications, *n* (%)	None	63/78 (80.8)	83/97 (85.6)	-
	Endocarditis	7/78 (9)	5/97 (5.2)	
	Osteoarticular	2/78 (2.6)	3/97 (3.1)	
	Abscess/myositis	4/78 (5.1)	3/97 (3.1)	
	Pulmonary embolisms	4/78 (5.1)	3/97 (3.1)	
	Embolisms CNS	1/78 (1.3)	4/97 (4.1)	
	Others	1/78 (1.3)	0/97	
Persistent bacteraemia, *n* (%)		8/80 (10)	13/100 (13)	0.26
Sepsis at diagnosis, *n* (%)		29 (32.6)	44 (38)	0.51
Early empirical therapy (before 24h), *n* (%)		70 (80.5)	113 (94.3)	0.005
Adequate empirical therapy, *n* (%)		68 (79.1)	103 (88)	0.12
Adequate empirical dose, *n* (%)		61 (71.8)	96 (89.7)	0.03
Adequate targeted therapy, *n* (%)		75 (94.9)	100 (95.2)	1
Adequate targeted dose, *n* (%)		72 (90)	96 (91.4)	0.94

*n*: total number of patients in that category. CNS: central nervous system.

**Table 3:** Adherence to the bundle.

	Pre-Pandemic Period	Pandemic Period	*p*
Adequate duration, *n* (%)	44/67 (65.7)	53/80 (66.3)	1
Echocardiogram, *n* (%)	65/81 (80.2)	80/103 (77.7)	0.8
Control blood cultures, *n* (%)	50/80 (62.5)	59/100 (59)	0.82
Compliance with all recommendations, *n* (%)	38 (50.7)	54 (54.5)	0.72

*n*: total number of patients in that category.

**Table 4:** Comparison of bacteraemia due to *Staphylococcus aureus* in patients with COVID-19 and patients without COVID-19 during the pandemic period.

		With COVID-19	Without COVID-19	*p*
Bacteria MSSA, *n* (%)		16 (94.1)	93 (92.1)	1
Acquisition, *n* (%)	Community	0	29 (28.7)	-
	Related to healthcare	3 (17.6)	21 (20.8)	
	Nosocomial	14 (82.4)	51 (50.5)	0.007
Source of respiratory infection, *n* (%)		9 (52.9)	16 (15.8)	0.001
Source of catheter infection, *n* (%)		4 (23.5)	21 (20.8)	0.948
14-day mortality, *n* (%)		11 (64.7)	20 (19.8)	<0.001
30-day mortality, *n* (%)		12 (70.6)	26 (26.3)	<0.001
Complications/death at 30 days, *n* (%)		12 (70.6)	34 (34.3)	0.005

*n*: total number of patients in that category. MSSA: methicillin-sensitive *S. aureus*.

## Data Availability

Data from the present study were reported to the PIRASOA program. Ministry of Health. Government of Andalucia. http://pirasoa.iavante.es/course/view.php?id=3&section=2 (accessed on 25 August 2024).

## References

[1] Minter D.J., Appa A., Chambers H.F., Doernberg S.B. (2023). Contemporary Management of *Staphylococcus aureus* Bacteremia—Controversies in Clinical Practice. Clin. Infect. Dis..

[2] Lam J.C., Stokes W. (2023). The Golden Grapes of Wrath—*Staphylococcus aureus* Bacteremia: A Clinical Review. Am. J. Med..

[3] Hindy J.R., Quintero-Martinez J.A., Lahr B.D., DeSimone D.C., Baddour L.M. (2023). *Staphylococcus aureus* bacteraemia and mortality: A population-based study in Olmsted County, Minnesota, from 2006 to 2020. Infect. Dis..

[4] Willekens R., Puig-Asensio M., Suanzes P., Fernández-Hidalgo N., Larrosa M.N., González-López J.J., Rodríguez-Pardo D., Pigrau C., Almirante B. (2021). Mortality in *Staphylococcus aureus* bacteraemia remains high despite adherence to quality indicators: Secondary analysis of a prospective cohort study. J. Infect..

[5] Abdollahi A., Nojomi M., Karimi Y., Ranjbar M. (2024). Mortality patterns in patients with *Staphylococcus aureus* bacteremia during the COVID-19 pandemic: Predictors and insights. Heliyon.

[6] Nambiar K., Seifert H., Rieg S., Kern W.V., Scarborough M., Gordon N.C., Kim H.B., Song K.-H., Tilley R., Gott H. (2018). Survival following *Staphylococcus aureus* bloodstream infection: A prospective multinational cohort study assessing the impact of place of care. J. Infect..

[7] Ikuta K.S., Swetschinski L.R., Robles Aguilar G., Sharara F., Mestrovic T., Gray A.P., Weaver N.D., Wool E.E., Han C., Hayoon A.G. (2022). Global mortality associated with 33 bacterial pathogens in 2019: A systematic analysis for the Global Burden of Disease Study 2019. Lancet.

[8] Lopez-Cortes L.E., Del Toro M.D., Galvez-Acebal J., Bereciartua-Bastarrica E., Farinas M.C., Sanz-Franco M., Natera C., Corzo J.E., Lomas J.M., Pasquau J. (2013). Impact of an Evidence-Based Bundle Intervention in the Quality-of-Care Management and Outcome of *Staphylococcus aureus* Bacteremia. Clin. Infect. Dis..

[9] Arientová S., Jícha Z., Beran O., Holub M. (2022). Decreased quality of care for *Staphylococcus aureus* bacteremia during the COVID-19 pandemic. BMC Infect. Dis..

[10] Böing C.W., Froböse N.J., Schaumburg F., Kampmeier S. (2023). Impact of the COVID-19 Pandemic on the Management of *Staphylococcus aureus* Bloodstream Infections in a Tertiary Care Hospital. Pathogens.

[11] Manna S., Baindara P., Mandal S.M. (2020). Molecular pathogenesis of secondary bacterial infection associated to viral infections including SARS-CoV-2. J. Infect. Public Health.

[12] Espinosa Pérez M., Fenoll R.G., Bayo S.M., Álvarez R.M.M., Millán V.F., Usón M.C.V., Ruiz M.P.P., Mainar J.M.V., Jiménez M.C.M., Paesa C.R. (2022). Impacto de la bacteriemia por *Staphylococcus aureus* en pacientes con COVID-19. Rev. Esp. Quim..

[13] Munro C., Zilberberg M.D., Shorr A.F. (2024). Bloodstream Infection in the Intensive Care Unit: Evolving Epidemiology and Microbiology. Antibiotics.

[14] Dar S., Erickson D., Manca C., Lozy T., Shashkina E., Kordalewska M., Mediavilla J.R., Chen L., Rojtman A., Kreiswirth B.N. (2023). The impact of COVID on bacterial sepsis. Eur. J. Clin. Microbiol. Infect. Dis..

[15] Serra N., Di Carlo P., Andriolo M., Mazzola G., Diprima E., Rea T., Anastasia A., Fasciana T.M.A., Pipitò L., Capra G. (2023). *Staphylococcus aureus* and Coagulase-Negative Staphylococci from Bloodstream Infections: Frequency of Occurrence and Antimicrobial Resistance, 2018–2021. Life.

[16] Haque O.I., Shameem M., Hashim W. (2023). Secondary infections in critically ill patients with COVID-19: A retrospective single-center study. Lung India.

[17] Mason E., Nsonwu O., Elmes J., Chudasama D., Pearson C., Hasan L., Hope R., Gerver S.M. (2024). Increased rates of hospital-onset *Staphylococcus aureus* bacteraemia in National Health Service acute trusts in England between June 2020 and March 2021: A national surveillance review. J. Hosp. Infect..

[18] Ruiz-Bastián M., Falces-Romero I., Ramos-Ramos J.C., de Pablos M., García-Rodríguez J., SARS-CoV-2 Working Group (2021). Bacterial co-infections in COVID-19 pneumonia in a tertiary care hospital: Surfing the first wave. Diagn. Microbiol. Infect. Dis..

[19] Nebreda-Mayoral T., Miguel-Gómez M.A., March-Rosselló G.A., Puente-Fuertes L., Cantón-Benito E., Martínez-García A.M., Muñoz-Martín A.B., Orduña-Domingo A. (2022). Bacterial/fungal infection in hospitalized patients with COVID-19 in a tertiary hospital in the Community of Castilla y León, Spain. Enferm. Infecc. Microbiol. Clin..

[20] Bauer K.A., Puzniak L.A., Yu K.C., Finelli L., Moise P., Watts J.A., Watts J.A., Gupta V. (2022). Epidemiology and outcomes of culture-positive bloodstream pathogens prior to and during the SARS-CoV-2 pandemic: A multicenter evaluation. BMC Infect. Dis..

[21] Adalbert J.R., Varshney K., Tobin R., Pajaro R. (2021). Clinical outcomes in patients co-infected with COVID-19 and *Staphylococcus aureus*: A scoping review. BMC Infect. Dis..

[22] Cusumano J.A., Dupper A.C., Malik Y., Gavioli E.M., Banga J., Berbel A.B., Nadkarni D., Obla A., Vasa C.V., Mazo D. (2020). *Staphylococcus aureus* Bacteremia in Patients Infected With COVID-19: A Case Series. Open Forum Infect. Dis..

[23] Asgill T.F., Stupart D. (2023). Nosocomial bacterial infections in Victoria decreased during the COVID-19 pandemic. J. Infect. Prev..

[24] Instituto de Estadística y Cartografía de Andalucía—Informe COVID-19 en Andalucía [Internet]. https://www.juntadeandalucia.es/institutodeestadisticaycartografia/badea/informe/datosaldia#79321.

[25] McCabe W.R., Jackson G.G. (1962). Gram-negative bacteriemia. Etiology and ecology. Arch. Lntern. Med..

[26] Gudiol F., Aguado J.M., Almirante B., Bouza E., Cercenado E., Domínguez M.Á., Gasch O., Lora-Tamayo J., Miró J.M., Palomar M. (2015). Diagnosis and treatment of bacteremia and endocarditis due to *Staphylococcus aureus*. A clinical guideline from the Spanish Society of Clinical Microbiology and Infectious Diseases (SEIMC). Enferm. Infecc. Y Microbiol. Clínica.

[27] Liu C., Bayer A., Cosgrove S.E., Daum R.S., Fridkin S.K., Gorwitz R.J., Kaplan S.L., Karchmer A.W., Levine D.P., Murray B.E. (2011). Clinical Practice Guidelines by the Infectious Diseases Society of America for the Treatment of Methicillin-Resistant *Staphylococcus aureus* Infections in Adults and Children. Clin. Infect. Dis..

[28] Cauhapé V., Lamy B., Lotte R., Touitou I., Boyer L., Contenti J., Parisot F., Ruimy R., Carles M., Courjon J. (2023). Lesson from the COVID-19 pandemic lockdown: A major change of hospital-diagnosed bacteremia epidemiology. Infect. Dis. Now.

[29] Morales-Cartagena A., Fernández-Ruiz M., Lalueza A., Lora-Tamayo J., San Juan R., López-Medrano F., Origüen J., Chaves F., Aguado J.M. (2018). Impact on mortality of adherence to evidence-based interventions in patients with catheter-related bloodstream infection due to methicillin-sensitive *Staphylococcus aureus*. Infect. Dis..

[30] Escrihuela-Vidal F., Kaasch A.J., Von Cube M., Rieg S., Kern W.V., Seifert H., Song K.-H., Liao C.-H., Tilley R., Gott H. (2023). Impact of adherence to individual quality-of-care indicators on the prognosis of bloodstream infection due to *Staphylococcus aureus*: A prospective observational multicentre cohort. Clin. Microbiol. Infect..

[31] Falces-Romero I., Bloise I., García-Rodríguez J., Cendejas-Bueno E. (2023). *Staphylococcus aureus* bacteremia in patients with SARS-CoV-2 infection. Med. Clin..

